# ENEA Reference Atmosphere Facility for Testing Radon and Daughters Measuring Equipment

**DOI:** 10.6028/jres.095.015

**Published:** 1990

**Authors:** G. Sciocchetti, F. Scacco, S. Tosti, P. G. Baldassini, E. Soldano

**Affiliations:** ENEA C.R.E. Casaccia C.P. 2400, 00100 Roma A.D., Italy

**Keywords:** calibration, radon and daughters measuring equipment, radon chamber, radon reference atmospheres

## Abstract

This paper gives the technical characteristics of the Italian Committee for Research and Development of Nuclear Energy and Alternative Energy (ENEA) radon chamber and the operational procedures developed for testing radon and daughters measuring equipment. Runs were carried out under different experimental conditions defined in terms of radon and daughter concentrations, equilibrium ratio (*F*-factor), and aerosol concentration and size distribution. Stable radon reference atmospheres with known equilibrium factors were obtained using standard aerosols.

## 1. Introduction

Starting in 1970, the Italian Committee for Research and Development of Nuclear Energy and Alternative Energy (ENEA) developed a research program on radon test facilities [1]. Experimental radon chambers were designed to produce reference exposure atmospheres containing traces of radon and thoron progeny.

A radon test chamber of 1 m^3^ was placed in operation in 1985 in the ENEA laboratory. The device allows experimental tests and calibration of radon and daughter measuring equipment under controlled climatic and aerosol conditions.

The testing of instruments used to measure radon decay products presents special problems because their accuracy of response is influenced by several factors, e.g., plate-out on surfaces, equilibrium factor, aerosol size distribution and concentration, and geometrical design and aerodynamical properties of the sampling devices. Instrument calibrations are usually carried out using reference alpha particle sources and air flow measurement standards.

The chamber that has been designed at ENEA provides reference radioactive atmospheres which tests the performance of both the sampling and detection subassembly of the instrument. Special requirements have to be fulfilled to maintain a stable particle concentration and size distribution. Investigations have been carried out to provide a range of radon daughter concentrations at different disequilibrium ratios in the chamber atmosphere. This paper briefly outlines the laboratory facilities, measuring methods, and technical equipment.

## 2. Description of ENEA Radon Test Facility

### 2.1 Criteria of Design and Applications

Procedural and technical aspects concerning laboratory calibration of radon and daughters measuring equipment have been discussed in the report of a group of experts of the Nuclear Energy Agency- of the Organization for Economic Cooperation and Development (OECD) [2]. Instruments used for the measurement of radon daughters need to be periodically calibrated against radioactive atmospheres of known characteristics. The following main parameters are used to characterize reference atmospheres:

- radon and daughters concentration;
- radon daughter disequilibrium ratios;
- unattached fraction;
- aerosol concentration and particle size distribution;
- climatic factors: relative humidity, pressure, and temperature.

The above parameters are also used to characterize atmospheres for thoron and its decay products as well as atmospheres for mixtures of radon and thoron decay products.

### 2.2 Technical Characteristics

**Figure 1:**
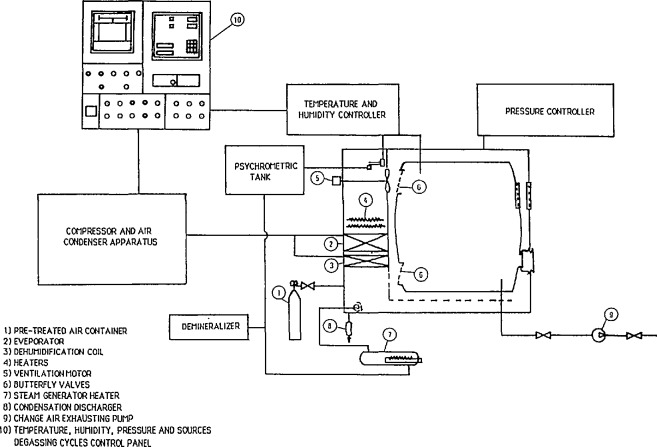
Diagram of the climatization apparatus of the radon test facility.

The ENEA facility consists of the following main components (fig. 1):

- cylindrical stainless steel chamber;
- climatization apparatus with automatic and manual control system;
- climatic parameters monitoring and data acquisition system;
- aerosol system;
- radon sources;
- radon and daughters monitoring devices.

A characteristic of this facility is that it can be operated in a static state and under two different dynamic conditions—recirculation and dynamic open circuit. During recirculation the air mass is taken from the front side of the chamber and reintroduced through the rear wall. Under dynamic open circuit conditions, the air mass taken from the chamber is continuously exhausted outside.

### 2.3 Chamber Climatic System

A special climatization system^1^,^2^ allows control of the following parameters without influencing the aerosol concentration and size distribution of the reference atmosphere:

- temperature range: from −35 to +60°C
- relative humidity range: from 10 to 95% in the temperature range from +5 to +60 °C with +4 °C dew point limitation;
- pressure range: from 700 to 1100 mBar.

The control of the operating conditions is fully automated. A microprocessor (programmer-controller) controls experimental cycles. A control panel is operated via a 10-key keyboard and displays set points or actual values (fig. 1).

## 3. Aerosol System

The aerosol system used is the Model Mage condensation nuclei generator for solid monodispersed particles.^3^ This device consists of a condensation nuclei source, a thermostated bubbler containing the substance to be vaporized, and a reheater. A carnauba wax solid aerosol is used as the test [3].

## 4. Particle Concentration and Size Distribution Monitor

The aerosol concentration and size distribution are determined using the TSI Aerodynamic Particle Sizer type APS 33B. The instrument operates at both high and very low particle concentrations and measures the aerodynamic equivalent diameter in the 0.5 to 15-*µ*m range. The instrument may be used for continuously monitoring the chamber atmosphere.

## 5. Experimental Tests at Dynamic Conditions

One of the objectives of the first experimental phase was to produce controlled atmospheres with a stable radon and daughters activity per unit volume at well defined concentration and size distribution of aerosols.

**Figure 2:**
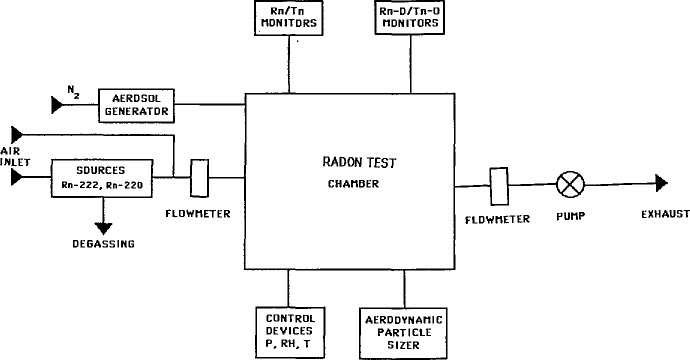
Diagram of the radon test facility.

Tests were carried out under dynamic conditions using the experimental apparatus shown in figure 2. A radium solution was used as the radon source. Pylon sources were also routinely used. The radon concentration was maintained constant by continuously bubbling air through the solution during tests. A monodispersed carnauba wax aerosol was continuously injected to maintain a stable concentration of condensation nuclei. Radon and radon daughters concentrations were monitored using both commercially available monitors and monitors designed at the ENEA laboratory.

## 6. Test Results

Runs were carried out using the radon chamber in the open circuit dynamic operating mode under the following experimental conditions:

- flow rates ranging from 11 to 31 L/min;
- particle size distribution with a maximum frequency of about 0.6 *µ*m.

Tests showed also that radon and daughters concentrations were uniform around the axis of the cylindrical volume of the chamber (useful volume). In the experimental runs, air samples were collected at a fixed point on the axis of the cylinder.

A first set of measurements was made in reference atmospheres using calibrated radon sources and monodispersed aerosols at controlled climatic conditions (temperature, relative humidity, and pressure). In the 11 to 31 L/min flow-rate range the measured radon concentrations varied from about 370 to 1110 Bq/m^3^ (10 to 30 pCi/L) and potential alpha energy concentrations from about 0.83 to 4.78 *µ*J/m^3^ (40 to 230 mWL).

The equilibrium factor (*F*-factor)^4^ values were in the 0.5 to 0,7 range. During experimental runs, stable conditions were obtained after a delay time ranging from 2 to 6 h, at flow rates, respectively, of 31 and 11 L/min.

Further experimental tests are in progress to obtain a larger range of equilibrium factor values at well defined aerosol concentrations and particle size distributions.

## Footnotes

1 The system was developed with the technical cooperation of Angelantoni Company (Perugia, Italy).

2 Certain commercial equipment, instruments, or materials are identified in this paper to specify adequately the experimental procedure. Such identification does not imply recommendation or endorsement by the National Institute of Standards and Technology, nor does it imply that the materials or equipment identified are necessarily the best available for the purpose.

3 Produced by the Lavoro Ambiente Company, Bologna, Italy.

4 The F-factor is the ratio of the equilibrium equivalent radon concentration *(EEC)* to the actual radon concentration (*c*) in the air: *F*=*EEC/c*.
